# PATIENT PORTAL MESSAGING IN THE CONTEXT OF DEMENTIA: A FIRST LOOK

**DOI:** 10.1093/geroni/igad104.0576

**Published:** 2023-12-21

**Authors:** Aleksandra Wec, Stephanie Nothelle, Kelly Gleason, Jennifer Wolff, Halima Amjad

**Affiliations:** Johns Hopkins Bloomberg School of Public Health, Baltimore, Maryland, United States; Johns Hopkins School of Medicine, Baltimore, Maryland, United States; Johns Hopkins, Baltimore, Maryland, United States; Johns Hopkins University, Baltimore, Maryland, United States; Johns Hopkins University School of Medicine, Baltimore, Maryland, United States

## Abstract

Primary care providers (PCPs) play an integral role in diagnosis and early management of dementia. Secure messaging through the patient portal is increasingly being used by patients to communicate with PCPs. Little is known about the nature of concerns raised by patients with dementia and their care partners when messaging PCPs following dementia diagnosis. We conducted a content analysis of 100 message threads initiated by patients and care partners with PCPs at an academic integrated health system during the twelve months following diagnosis of dementia. Message threads ranged in length from two to eleven messages and included 337 unique messages from 79 distinct accounts, sent between 01/03/2020 and 01/20/2022. Of 100 message threads, 89 were sent by care partners and 11 were sent by patients. Threads often focused on medications. Of 36 threads mentioning medications, 12 discussed dementia-specific medications. Other common dementia-specific topics included behavioral and/or cognitive symptoms of dementia (22/100) and sleep disturbance (8/100). Safety concerns related to falls, wandering, or driving were also common (15/100). Care partners raised issues related to their own needs or wellbeing (e.g., caregiving stress, need for caregiver education) (15/100 threads) and messaged to coordinate care between the PCP and other providers (26/100). Findings demonstrate care partners commonly message on behalf of persons with dementia and use the portal to address dementia-related care needs. Identifying issues raised via messaging offers insight into resources and strategies for PCPs to effectively and efficiently address common concerns after dementia diagnosis, including strategies that leverage the patient portal.

